# Quantification of Phospholipids Classes in Human Milk

**DOI:** 10.1007/s11745-013-3825-z

**Published:** 2013-08-28

**Authors:** Francesca Giuffrida, Cristina Cruz-Hernandez, Brigitte Flück, Isabelle Tavazzi, Sagar K. Thakkar, Frédéric Destaillats, Marcel Braun

**Affiliations:** Nestlé Product Technology Centre, Konolfingen, Switzerland

**Keywords:** Phosphatidylinositol (Ptdlns), Phosphatidylethanolamine (PtdEtn), Phophatidylserine (PtdSer), Phosphatidylcholine (PtdCho), Sphingomyelin (CerPCho), Human milk

## Abstract

Phospholipids are integral constituents of the milk fat globule membranes and they play a central role in infants’ immune and inflammatory responses. A methodology employing liquid chromatography coupled with evaporative light scattering detector has been optimized and validated to quantify the major phospholipids classes in human milk. Phospholipids were extracted using chloroform and methanol and separated on C18 column. Repeatability, intermediate reproducibility, and recovery values were calculated and a large sample set of human milk analyzed. In human milk, phospholipid classes were quantified at concentrations of 0.6 mg/100 g for phosphatidylinositol; 4.2 mg/100 g for phosphatidylethanolamine, 0.4 mg/100 g for phosphatidylserine, 2.8 mg/100 g for phosphatidylcholine, and 4.6 mg/100 g for sphingomyelin. Their relative standard deviation of repeatability and intermediate reproducibility values ranging between 0.8 and 13.4 % and between 2.4 and 25.7 %, respectively. The recovery values ranged between 67 and 112 %. Finally, the validated method was used to quantify phospholipid classes in human milk collected from 50 volunteers 4 weeks postpartum providing absolute content of these lipids in a relatively large cohort. The average content of total phospholipids was 23.8 mg/100 g that corresponds to an estimated mean intake of 140 mg phospholipids/day in a 4-week old infant when exclusively breast-fed.

## Introduction

Human milk is advocated as the optimal form of nourishment for infants during the first 6 months of life [1] and among its macronutrients, the lipid fraction is crucial, representing almost 50 % of their daily calories [2]. Lipids are secreted in milk in the form of fat globules and are mainly composed of triacylglycerols (~98 % of total lipids) surrounded by a structural membrane composed of phospholipids (PL), cholesterols, enzymes, proteins, glycosphingolipids and glycoproteins [3]. In the gastric tract, dietary as well as endogenous bile PL contribute to solubilization of lipid digestion products for efficient absorption and transport [4]. Furthermore, PL are involved in immunity and inflammatory responses [5], in neuronal signaling and they seem to attenuate the effects of age-related diseases [6]. Given the important role of PL in infant nutrition, data related to their absolute amount in human milk are extremely important. Due to the amphiphilic nature of PL, their quantitative analysis is not straightforward. In the past decade the most frequently used technique for characterization of PL profile in maternal milk was the thin layer chromatography [7]. This technique has some advantages, since it is inexpensive and simple, but it is usually time consuming and not robust enough for routine analyses of large numbers of samples [8]. An alternative technique used for the PL analysis in human milk is the ^31^P nuclear magnetic resonance (^31^P-NMR) [9]. This technique requires low amounts of sample and it is very selective for detecting only compounds that contain phosphorous group. However, ^31^P-NMR technique is very expensive and it requires highly qualified operators, making it inappropriate for the analyses of a large set of samples. The high performance liquid chromatography (HPLC) method [10, 11] coupled with evaporative light scattering detector (ELSD) is a common technique for PL quantification. HPLC coupled with mass spectrometer (MS) has also been used extensively for both characterization and quantification of PL in several biological matrices [12–17]. However, only a few articles on PL quantification in human milk have been published [3, 9, 10, 17–19], mainly based on total organic phosphorus determination with a prior separation of PL and neutral lipids, thin layer chromatography, ^31^P NMR and HPLC-ELSD. Among these techniques, the HPLC-ELSD is the most suitable for analyses of a large set of samples and in this study a chloroform free HPLC-ELSD method to quantify the most abundant milk PL classes, i.e., Ptdlns, PtdSer, PtdCho, PtdEtn and CerPCho in maternal milk was developed and validated. The sample weight was adjusted in order to reduce the amount of solvents used during the extraction procedure. To correct the loss of analyte during sample preparation, phosphatidylglycerol (PtdGro), a PL of synthetic origin, was used as an internal standard. The method was applied to a large maternal milk sample set in order to determine the absolute content of PL providing a new insight into infant lipid nutrition.

## Experimental

### Materials

Methanol, acetonitrile, chloroform, potassium chloride, ammonium formate, and PtdGro were purchased from Sigma–Aldrich (Buchs, Switzerland). The certified milk lecithin was provided by Spectral Service GmbH, Köln, Germany.

### Methods

#### Human Milk Collection

The study took place at the National University of Singapore. The protocol and collection of human milk was reviewed and approved by the local ethical committee of Singapore. The study was registered in ClinicalTrial.gov (NCT01805011).

Volunteer mothers of term infants, who were apparently healthy and non smokers (*n* = 50; 31.1 ± 3.1-year old) provided breast milk samples (approximately 30 mL; 4 weeks postpartum). Samples were collected after full expression from one breast using a milk pump and while the baby was being fed on the other breast. We made all efforts to collect complete feed that included fore-milk, mid-milk and hind-milk as a representation of one feed and to avoid within feed variation of lipid content. An approximately 30-mL aliquot was separated in a conical polypropylene tube for this study and the rest was fed to the infant. Samples collected for research were stored at −80 °C until analysis.

#### Phospholipid Quantification

PL classes were separated by normal-phase HPLC using 2 Nucleosil 50-5, 250 × 3 mm, 5 μm (Macherey–Nagel, Easton, USA) equipped with pre-column Nucleosil 50-5, 8 × 3 mm, 5 μm (Macherey–Nagel, Easton, USA) as previously described [20]. The chromatography system consisted of an Agilent 1,200 module (Agilent Technologies, Basel, Switzerland) and an in-line 385-ELS evaporative light scattering detector module (Agilent Technologies, Basel, Switzerland). All chromatography was performed at 55 °C. Solvent A contained ammonium formate 3 g/L and solvent B of acetonitrile/methanol (100/3 vol/vol). Gradient conditions for PL analysis were as follows: time = 0 min 1 % solvent A; time = 19 min 30 % solvent A; time = 21 min 30 % solvent A; time = 24 min 1 % solvent A; with a flow rate 1 mL/min. Injection volume was 0.01 mL. Data were collected and processed using Agilent Chem. Station software. The best signal and resolution was achieved at the following ELSD conditions: evap = 90 °C; neb = 40 °C, flow rate of N_2_ = 1 L/min. All analyses were performed in duplicate. For the PL classes quantification by ^31^P NMR, the samples were sent to an independent laboratory (Spectral Service, Köln, Germany).

#### Extraction and Purification of Phospholipids

Phospholipids were extracted according to modified Folch extraction [21]. Briefly, 250 mg of maternal milk was weighed into a test tube with a screw cap and mixed with 250 mg of water and 9.5 mL of chloroform/methanol (2/1 vol/vol). After precise addition of 10 μL of PtdGro internal standard solution (5 mg/mL), the sample solution was put into ultrasonic bath at 40 °C for 15 min. After centrifugation (1,000 RCF, relative centrifugal force, for 10 min), the sample solution was filtered through 0.2 μm PTFE filters into glass tubes using a vacuum manifold and elut reservoirs. The filtrate was mixed with 2 mL of potassium chloride solution (8.8 g/L) and centrifuged (1,000 RCF for 10 min). The organic phases were quantitatively transferred into Extrelut vials and solvents evaporated to dryness under a nitrogen flow at 40 °C. The residual lipids were redissolved in 150 μL of chloroform/methanol (9/1 vol/vol), filtered through 4-mm PVDF membrane filters into conical auto sampler vials and analyzed by HPLC-ELSD.

### Method Validation

Method validation was performed to assess the linearity, limit of quantification (LOQ), trueness and precision.

*Linearity; limit of quantification (LOQ)* The linearity of the method was assessed by analyzing six different concentrations of standard solutions of milk reference material covering ranges from 1.5 to 7.6 mg/100 g for Ptdlns, 3.7 to 7.31 mg/100 g for PtdEtn, 2.4 to 47.9 mg/100 g for PtdSer, 4.0 to 80.3 mg/100 g for PtdCho, 3.6 to 71.5 mg/100 g for CerPCho, and 2.5 to 49.6 mg/100 g for PtdGro, respectively. A separate calibration curve for each PL family was made. The calibration curves were plotted as peak areas of PL (*y*) vs. concentrations of the standard solutions (*x*). The LOQ was defined as the lowest validated concentration.

*Trueness* Recovery of added certified milk lecithin reference material was studied at three levels. 111 mg of certified reference material were accurately weighed in 10-mL volumetric flasks and mixed with 10 mL of chloroform/methanol (9/1 vol/vol). Aliquots of 10, 20 and 30 μL certified reference material solution were added to the sample. To further verify the accuracy of the HPLC-ELSD method the same milk samples were analyzed internally by HPLC-ELSD and by ^31^P NMR in an independent laboratory and a *t* test, i.e., two-sample assuming unequal variances, was applied to compare PL family concentrations measured.

*Precision* The precision of the method was evaluated by calculating the repeatability (r) and the intermediate reproducibility (iR). Repeatability represents the variability of independent results obtained in the same laboratory, with the same analyst, on the same equipment, in a short interval of time. Intermediate reproducibility represents the variability of independent results obtained in the same laboratory, on different days, with the same analyst, different calibrations, and same equipment. Repeatability and intermediate reproducibility were calculated by analyzing spiked samples in duplicate, on six different days, by the same analyst, with the same equipment and with different solution preparations. All results were evaluated using Q-Stat software (Nestlé, Switzerland).

## Results and Discussion

### Method Optimization

**Fig. 1:**
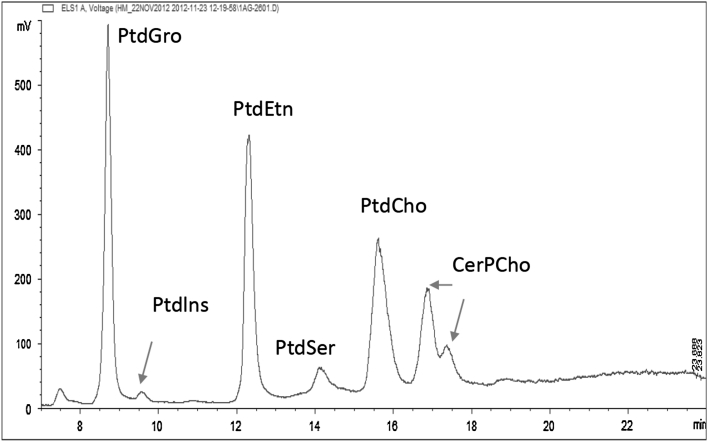
Typical HPLC-ELSD chromatogram of phosphatidyinositol (Ptdlns), phosphatidylethanolamine (PtdEtn), phosphatidylserine (PtdSer), phosphatidylcholine (PtdCho), sphingomyelin (CerPCho) in milk reference material

The aim of this study was to develop an HPLC-ELSD method to quantify the most abundant PL classes, i.e., Ptdlns, PtdEtn, PtdSer, PtdCho, and CerPCho, in human milk. Different columns, solvents and gradients were tested in this study (Table 1). The Nucleosil 50-5 column had superior resolution when ammonium formate and acetonitrile with methanol were used. Figure 1 shows a chromatogram of the milk reference material. The PL classes Ptdlns, PtdEtn, PtdSer, PtdCho and CerPCho were well separated from each other and from the internal standard (PtdGro) with the above described HPLC-ELSD conditions. The chronological elution order was PtdGro, Ptdlns, PtdEtn, PtdSer, PtdCho and CerPCho and PL classes were eluted within 25 min.

**Table 1:** Chromatographic condition tested in this study

Column	Solvents	Reference
Silica column, 150 × 3 mm, 3 μm (Phenomenex, Torrance, CA)	A:1 M formic acid, neutralized to pH3 with triethylamine B: chloroform C: methanol	[21]
Hypersil gold silica, 200 × 2.1 mm, 1.9 μm (Thermo Scientific, Wohlen, Switzerland)	A: Acetonitrile/50 mM ammonium acetate buffer, pH 5.6 (95:5) B: Acetonitrile/50 mM ammonium acetate buffer, pH 5.6 (50:50)	[13]
Nucleosil 50-5, 250 × 3 mm, 5μ (Macherey–Nagel, Easton, USA)	A: 50 mM ammonium formate B: acetonitrile/methanol (100/3)	[20]
Polaris 3, 250 × 2 mm (Agilent Technologies, Basel, Switzerland)	A: 50 mM ammonium formate B: acetonitrile/methanol (100/3)	–

### Method Validation

**Fig. 2:**
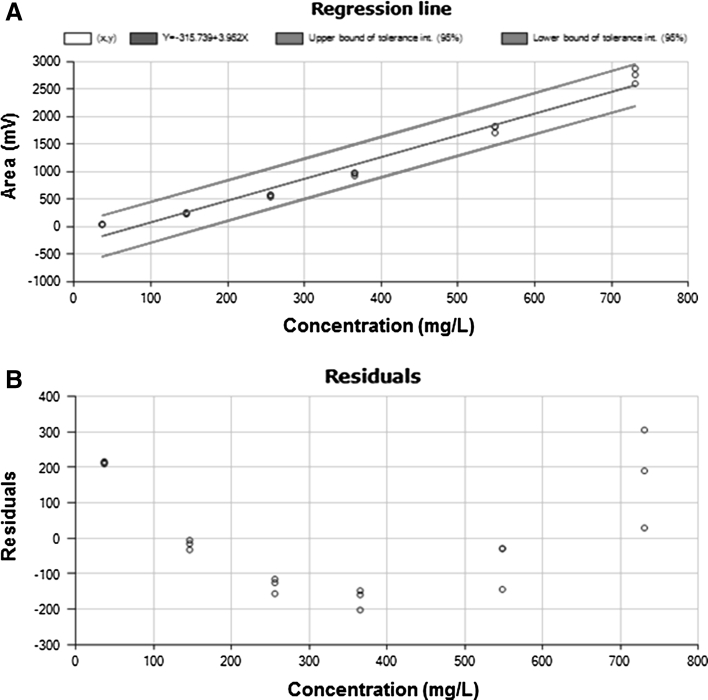
Example of standard calibration plot (**a**) and residual (**b**) of phosphatidylethanolamine (PtdEtn). Various concentrations of milk lecithin reference material were freshly made and injected over 3 days

*Linearity* Previous studies [22–25] reported that the ELSD response is not proportional to the amount of phospholipids eluting from the column. Therefore, calibration curves are required to quantify the separated components. In order to establish the best regression model to quantify phospholipids, the response (at least in triplicate) of six concentration levels of milk lecithin reference material covering a range from 1.5 to 7.6 mg/100 g for Ptdlns, 3.7 to 7.31 mg/100 g for PtdEtn, 2.4 to 47.9 mg/100 g for PtdSer, 4.0 to 80.3 mg/100 g for PtdCho, 3.6 to 71.5 mg/100 g for CerPCho, and 2.5 to 49.6 mg/100 g for PtdGro, respectively were assessed. Figure 2 shows an example of calibration plot and residual obtained for PtdEtn, similar behavior was observed for the other PL classes. The amount of each PL family was calculated from a second degree polynomial regression model (*y* = *ax* ^2^ + *bx* + *c*), and the heterogeneity of variance, i.e., dispersion of results at high concentrations (Fig. 2b), indicated the inaccuracy of a non-weighted model to evaluate the results. Therefore, a weighted second degree polynomial regression model, meaning that calibration points were weighted by the factor 1/amount^2^, was used to quantify PL classes. In order to improve the accuracy of quantification of PL classes separated by using a gradient HPLC-ELSD, the calibration curve containing the internal standard was injected every 20 samples, as a consequence, the quantification was performed by considering the average of the calibration curves injected before and after the sequence of 20 samples.

*Limit of Quantification* The LoQ was considered as the lowest validated concentration, i.e., 0.1 ng for Ptdlns, 0.4 ng for PtdEtn, 0.3 ng for PtdSer and PtdCho and 0.5 ng for CerPCho injected on column.

*Recovery* The trueness of the method was evaluated by spiking human milk samples with certified reference materials. Recovery was calculated by analyzing spiked samples in duplicate, on six different days, by the same analyst and with the same equipment. Recovery values (Rec) were compared with reference values. A *t* test was performed to check if recovery was significantly different from 100 %. The recovery ranged between 67 and 112 % (Table 2) and it was not significantly different from 100 %, except for Ptdlns. In addition, to further verify the accuracy of the HPLC-ELSD method, the same milk samples were analyzed by HPLC-ELSD and by ^31^P NMR and a *t* test, i.e., two-sample assuming unequal variances, was applied to compare PL family concentrations measured. PL family concentrations obtained by HPLC-ELSD and ^31^P NMR and the calculated *p* values are shown in Table 3. The *p* values were higher than 5 % when comparing the PL concentrations, therefore there was no difference in phospholipid concentrations determined by using the two analytical methods.

**Table 2:** Median, recovery (Rec), standard deviation of repeatability (SD(r)), relative standard deviation of repeatability (CV(r)), standard deviation of intermediate reproducibility (SD(iR)), and relative standard deviation of intermediate reproducibility (CV(iR)) of phospholipids in spiked human milk

Analyte	Added amount	Median	Rec %	Rec = 100 %	SD (r)	CV (r) %	SD (iR)	CV (iR) %
Ptdlns	1.9	1.3	71	N	0.1	9.5	0.1	10.2
	0.9	0.6	67	N	0.1	13.4	0.2	25.7
PtdEtn	18.0	16.9	94	Y	0.1	0.8	0.5	7.7
	9.1	7.9	87	Y	0.2	2.5	0.2	8.2
	4.2	4.2	101	Y	0.25	5.9	0.29	6.8
PtdSer	12.9	12.2	95	Y	0.4	3.2	0.7	5.9
	6.5	6.3	96	Y	0.02	5.4	0.09	24.2
	3.5	2.8	86	Y	0.4	12.5	0.6	22.3
	–	0.4	–	–	0.02	5.4	0.09	24.2
PtdCho	19.8	19.5	99	Y	0.2	0.9	0.4	5.7
	10.0	9.6	96	Y	0.2	2.4	0.3	8.8
	2.5	2.8	107	Y	0.04	1.4	0.15	5.7
CerPCho	17.6	19.8	112	Y	0.4	1.8	0.4	2.4
	6.6	6.2	94	Y	0.20	3.2	0.43	6.9
	4.4	4.6	105	Y	0.3	6.7	0.3	6.2

Results are expressed in mg/100 g of product

Phosphatidylinositol (Ptdlns), phosphatidylethanolamine (PtdEtn), phosphatidylserine (PtdSer), phosphatidylcholine (PtdCho), sphingomyelin (CerPCho)

* Y = the recovery was 100 %

**Table 3:** Phospholipids concentrations in milk products analyzed by ^31^P NMR and by HPLC-ELSD

Analyte	^31^P NMR	HPLC/ELSD	*P* value
Ptdlns	0.14	0.12	0.87
PtdEtn	0.35	0.38	0.91
PtdCho	0.37	0.38	0.98
CerPCho	0.29	0.27	0.91

Results are expressed in g/100 g of product

Phosphatidylinositol (Ptdlns), phosphatidylethanolamine (PtdEtn), phosphatidylcholine (PtdCho), sphingomyelin (CerPCho)

*Repeatability (r)* *and intermediate* *Reproducibility (iR)* The precision of the method was evaluated by calculating the simple repeatability and the intermediate reproducibility. Standard deviation of repeatability (SD(r)) and intermediate reproducibility (SD(iR)), and relative standard deviation of repeatability (CV(r)) and intermediate reproducibility (CV(iR)) are listed in Table 2. CV(r) and CV(iR) values ranged between 0.8 and 13.4 % and between 2.4 and 25.7 %, respectively. Values of 20 % for CV(r) and of 30 % for CV(iR) were considered acceptable with respect to analytical measurement of PL by HPLC-ELSD detector. This method was not suitable for the absolute quantification of Ptdlns at concentration lower than 0.9 mg/100 g, showing CV(r) and CV(iR) values higher than 25 %.

### Phospholipids Quantification in Human Milk

**Fig. 3:**
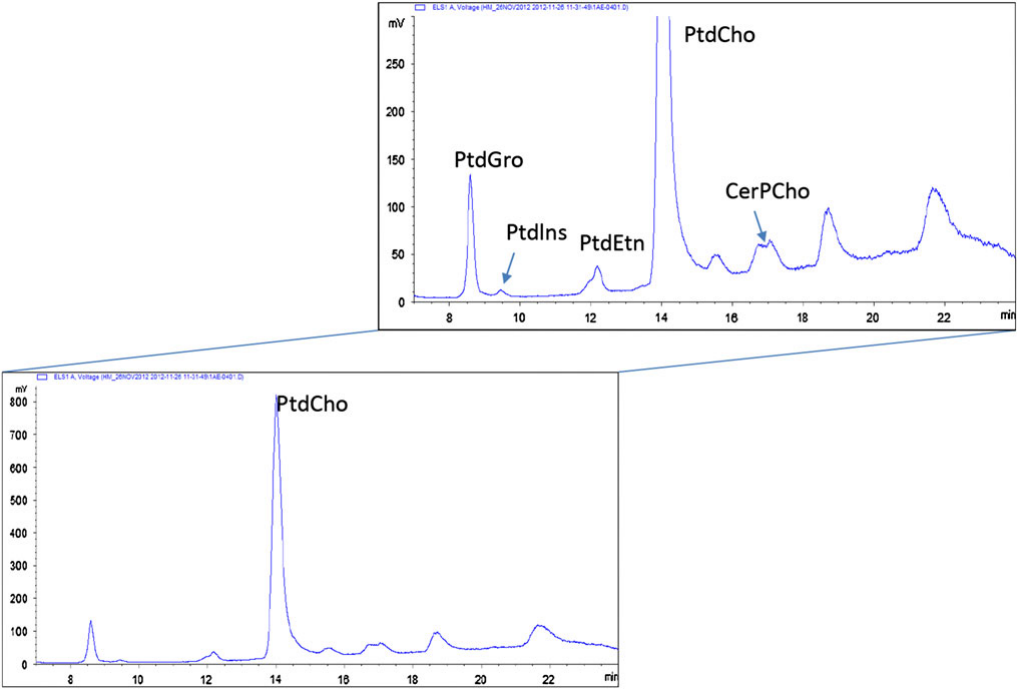
Typical HPLC-ELSD chromatogram of phosphatidylinositol (Ptdlns), phosphatidylethanolamine (PtdEtn), phosphatidylserine (PtdSer), phosphatidylcholine (PtdCho), sphingomyelin (CerPCho) in human milk

The validated HPLC-ELSD method has been used to analyze human milk collected 4 weeks postpartum (Fig. 3). As observed for the certified reference material, peak shape and resolution were not optimal for PtdSer. The separation of one PL family can create multiple peaks or peak shoulders, as the structure of individual species can be different depending on fatty acid moieties. In particular, the CerPCho resulted in two distinct peaks, probably due to the heterogeneity of acyl residues [26] or the presence/absence of multiple hydroxyl groups [27]. The content of Ptdlns ranged between 0.9 and 2.3 mg/100 g, PtdEtn between 3.1 and 11.8 mg/100 g, PtdSer between 1.0 and 1.9 mg/100 g, PtdCho between 3.2 and 9.6 mg/100 g and CerPCho between 4.7 and 12.8 mg/100 g (Table 4). In full expressed human milk collected 4 weeks postpartum CerPCho was the most abundant class followed by PtdEtn, PtdCho, PtdSer and Ptdlns. Previous studies [3, 9, 18] showed PtdCho being in higher proportion than PtdEtn, suggesting that in our samples lysophosphatidylethanolamine is probably quantified together with PtdEtn. We did not measure minor constituents such as lysophosphatidylcholine, which may contribute only with small amounts to the infant’s diet.

**Table 4:** Phospholipids concentration in human milk samples (*N* = 50) collected 4 weeks after delivery and expressed in absolute values (mg of phospholipids per 100 g of human milk)

Phospholipids	Phospholipids in human milk (*N* = 50, 4 weeks after birth)					
	Mean	in mg per 100 g of human milk				
		% in class	VAR	SD	Min	Max
Ptdlns	1.1	4.6	1.2	0.3	0.9	2.3
PtdEtn	6.8	28.6	34.7	1.9	3.1	11.8
PtdSer	1.4	5.9	1.5	0.3	1.0	1.9
PtdCho	6.0	25.2	18.1	1.3	3.2	9.6
CerPCho	8.5	35.7	29.9	1.7	4.7	12.8
Total	23.8	100	114.5	3.4	12.9	38.4

VAR and SD stand for variance and standard deviation

* Ptdlns* Phosphatidylinositol, *PtdEtn* phosphatidylethanolamine, *PtdSer* phosphatidylserine, *PtdCho* phosphatidylcholine,* CerPCho* sphingomyelin

Several studies [28–30] have recognized the importance of PL for infant growth, therefore PL may be of particular interest as functional ingredients, however, optimal dose and specific PL class have to be selected. From this study, it can be estimated that the mean intake of total phospholipids per day in infants 4 weeks old is about 140 mg when the infant is fed exclusively with human milk. These findings are in agreements with previous ones [9, 18, 19] which reported a mean intake of total phospholipids per day of 109, 126 and 150 mg, respectively. This estimation is based on the assumption that mean volume of human milk consumed at this age is 600 mL/day [31].

## Conclusions

In this study a HPLC-ELSD procedure to quantify phospholipids in human milk has been established and validated. This method has the advantages of robustness for the quantification of PL in maternal milk comparing to the existing methods. The established method was applied to analyze a large number of human milk samples demonstrating its applicability for large clinical trials. In addition the use of internal standard allowed correcting the loss of analyte during sample preparation. Further investigation is needed in order to determine the PL molecular species. As previously demonstrated [19, 22, 32], LC–MS would be the most appropriate technique for identifying them, on the other hand due to the dependency of the MS response and therefore peak area intensity on the acyl chain length and number of unsaturation, the quantification of PL molecular species requires the use of pure standards which are not always available. Finally, the variation in the concentration of PL over the different stages of lactations as well as the biological functions of PL during early development of infants should be further investigated.

## Abbreviations

^31^P NMR: ^31^P nuclear magnetic resonance
CerPCho: Sphingomyelin
CV(iR): Relative standard deviation of intermediate reproducibility
CV(r): Relative standard deviation of repeatability
ELSD: Evaporative light scattering detector
HPLC: High performance liquid chromatography
iR: The intermediate reproducibility
LOQ: Limit of quantification
MS: Mass spectrometer
PL: Phospholipids
PtdCho: Phosphatidylcholine
PtdEtn: Phosphatidylethanolamine
PtdGro: Phosphatidylglycerol
Ptdlns: Phosphatidylinositol
PtdSer: Phophatidylserine
R: Repeatability
Rec: Recovery
SD(iR): Standard deviation of intermediate reproducibility
SD(r): Standard deviation of repeatability
